# Study protocol for examining job strain as a risk factor for severe unipolar depression in an individual participant meta-analysis of 14 European cohorts

**DOI:** 10.12688/f1000research.2-233.v2

**Published:** 2014-02-26

**Authors:** Ida E. H. Madsen, Harald Hannerz, Solja T. Nyberg, Linda L. Magnusson Hanson, Kirsi Ahola, Lars Alfredsson, G. David Batty, Jakob B. Bjorner, Marianne Borritz, Hermann Burr, Nico Dragano, Jane E. Ferrie, Mark Hamer, Markus Jokela, Anders Knutsson, Markku Koskenvuo, Aki Koskinen, Constanze Leineweber, Martin L. Nielsen, Maria Nordin, Tuula Oksanen, Jan H. Pejtersen, Jaana Pentti, Paula Salo, Archana Singh-Manoux, Sakari Suominen, Töres Theorell, Salla Toppinen-Tanner, Jussi Vahtera, Ari Väänänen, Peter J. M Westerholm, Hugo Westerlund, Eleonor Fransson, Katriina Heikkilä, Marianna Virtanen, Reiner Rugulies, Mika Kivimäki, for the IPD-Work Consortium

**Affiliations:** 1 National Research Centre for the Working Environment, Copenhagen, DK-2100, Denmark; 2 Finnish Institute of Occupational Health, Helsinki, FI-00250, Finland; 3 Stress Research Institute, Stockholm University, Stockholm, SE-171 77, Sweden; 4 Institute of Environmental Medicine, Karolinska Institutet, Stockholm, SE-171 77, Sweden; 5 Centre for Occupational and Environmental Medicine, Stockholm County Council, Stockholm, SE-104 22, Sweden; 6 Department of Epidemiology and Public Health, University College London, London, GB-WC1E 6BT, UK; 7 Centre for Cognitive Ageing and Cognitive Epidemiology, University of Edinburgh, Edinburgh, GB-EH8 9JZ, UK; 8 Alzheimer Scotland Dementia Research Centre, University of Edinburgh, Edinburgh, GB-EH8 9JZ, UK; 9 Department of Occupational and Environmental Medicine, Bispebjerg University Hospital, Copenhagen, DK-2400, Denmark; 10 Federal Institute for Occupational Safety and Health (BAuA, Berlin, DE- 10317, Germany; 11 Department of Medical Sociology, University of Düsseldorf, Düsseldorf, DE-40225, Germany; 12 School of Community and Social Medicine, University of Bristol, Bristol, GB-BS8 2PS, UK; 13 Institute of Behavioral Sciences, University of Helsinki, Helsinki, FI-00014, Finland; 14 Department of Health Sciences, Mid Sweden University, Sundsvall, SE-851 70, Sweden; 15 Department of Public Health, University of Helsinki, Helsinki, FI-00014, Finland; 16 Department of Public Health and Clinical Medicine, Occupational and Environmental Medicine, Umeå University, Umeå, SE-901 85, Sweden; 17 The Danish National Centre for Social Research, Copenhagen, DK-1052, Denmark; 18 Department of Psychology, University of Turku, Turku, FI- 20014, Finland; 19 Inserm U1018, Centre for Research in Epidemiology and Population Health, Villejuif, F-94807, France; 20 Folkhälsan Research Center, Helsinki, FI-00290, Finland; 21 Nordic School of Public Health, Göteborg, SE-402 42, Sweden; 22 Department of Public Health, University of Turku, Turku, FI-20014, Finland; 23 Turku University Hospital, Turku, FI-20520, Finland; 24 Occupational and Environmental Medicine, Uppsala University, Uppsala, SE- 751 85, Sweden; 25 School of Health Sciences, Jönköping University, Jönköping, SE- 553 18, Sweden; 26 Department of Public Health and Department of Psychology, University of Copenhagen, Copenhagen, DK-1353, Denmark

## Abstract

**Background: **Previous studies have shown that gainfully employed individuals with high work demands and low control at work (denoted “job strain”) are at increased risk of common mental disorders, including depression. Most existing studies have, however, measured depression using self-rated symptom scales that do not necessarily correspond to clinically diagnosed depression. In addition, a meta-analysis from 2008 indicated publication bias in the field.

**Methods: **This study protocol describes the planned design and analyses of an individual participant data meta-analysis, to examine whether job strain is associated with an increased risk of clinically diagnosed unipolar depression based on hospital treatment registers.  The study will be based on data from approximately 120,000 individuals who participated in 14 studies on work environment and health in 4 European countries. The self-reported working conditions data will be merged with national registers on psychiatric hospital treatment, primarily hospital admissions. Study-specific risk estimates for the association between job strain and depression will be calculated using Cox regressions. The study-specific risk estimates will be pooled using random effects meta-analysis.

**Discussion: **The planned analyses will help clarify whether job strain is associated with an increased risk of clinically diagnosed unipolar depression. As the analysis is based on pre-planned study protocols and an individual participant data meta-analysis, the pooled risk estimates will not be influenced by selective reporting and publication bias. However, the results of the planned study may only pertain to severe cases of unipolar depression, because of the outcome measure applied.

## Introduction

Unipolar depression is prevalent and incurs substantial costs for the individuals affected and society at large ^1, 2^. The disorder is thought to develop in a complex interplay of biological, psychological and social factors ^3– 5^. Following the diathesis-stress framework ^6^, etiological determinants throughout the life-course may affect vulnerability to depression or act as triggering factors.

According to the job strain model ^7^, a psychosocial work environment characterized by high psychological demands and low control may result in stress-reactions and lead to adverse health outcomes. Accordingly, job strain has been linked to several health conditions, including coronary heart disease ^8^ and unipolar depression ^9, 10^. There are, however, indications of publication bias in the field, suggesting that the published literature may be biased towards studies showing stronger associations between job strain and depression ^10^. In addition, many previous studies on job strain and depression have applied outcome measures with uncertain diagnostic validity, for example self-reported symptoms ^9^. Hence, the applicability of these findings to clinically diagnosed depression is uncertain ^9^. In this study protocol, we set out our plan to obtain data from 14 European cohort studies on work environment and health, and to examine the association between job strain and subsequent first hospitalisation due to a diagnosis of unipolar depression. The purpose of this planned project is to examine whether stressful working conditions characterized by high psychological demands and low control, i.e. job strain ^7^, are a risk factor for the development of unipolar depressive disorder. We hypothesize that individuals experiencing job strain are more likely to become hospitalized with a diagnosis of unipolar depression than individuals without job strain. Furthermore we aim to explore whether the association between job strain and depression is similar across strata of sex, age and socioeconomic status (SES).

## Data

The IPD-Work Consortium combines data from a number of European work environment studies. For the analyses on job strain and depression we include data from 14 studies that are linked with hospital admission registers including psychiatric admissions. These studies encompass a total of approximately 120,000 individuals. Table 1 gives an overview of the included studies.

**Table 1.** Overview of included studies.

Study ^a^	Country	Year of baseline	Estimated N participants ^b^
**COPSOQ I**	Denmark	1997	1,724
**COPSOQ II**	Denmark	2004–2005	3,326
**DWECS 2000**	Denmark	2000	5,463
**DWECS 2005**	Denmark	2005	4,021
**FPS**	Finland	2000	47,373
**HeSSup**	Finland	1998	16,345
**IPAW**	Denmark	1996–97	2,022
**PUMA**	Denmark	1999	1,731
**SLOSH 2006**	Sweden	2006	5,104
**SLOSH 2008**	Sweden	2008	5,895
**Still working**	Finland	1986	9,129
**Whitehall II**	United Kingdom	1985–1988	10,250
**WOLF-N**	Sweden	1996–98	4,678
**WOLF-S**	Sweden	1992–95	5,653
**Total**			122,714

^a^Study acronyms: COPSOQ: Copenhagen Psychosocial Questionnaire Study, DWECS: Danish Work Environment Cohort Study, FPS: Finnish Public Sector study, HeSSUP: Health and Social support Study, IPAW: Intervention Project on Absence and Well-being, PUMA: Burnout, Motivation and Job Satisfaction study, SLOSH: Swedish Longitudinal Occupational Survey of Health, WOLF: Work, Lipids, Fibrinogen (N = Norrland, S = Stockholm).

^b^Estimates based on previous analyses or baseline data.

In each study, data on psychiatric hospital treatment are available through national registers ^11– 14^. Most registers include both inpatient and outpatient treatments. Outpatient data are available since 1995 in the Danish register, since 2001 in the Swedish register, since 1998 in the Finnish register and since 2003 in the UK register. To maximize the number of cases, these data are included when available.

## Study population & design

The study is designed as a prospective cohort study. Participants will be included if they are employed at baseline of the respective study and have provided data on job strain, sex, age, cohabitation and socioeconomic status (SES). To ensure a prospective design we exclude all individuals with a hospital-based diagnosis of unipolar depression before or at baseline. Data are analysed using a two-step individual participant data meta-analysis; i.e. we first obtain study-specific risk estimates using harmonised exposure and outcome data and then combine these estimates using meta analytic techniques ^15^. The study-specific risk estimates are calculated using Cox regressions analysis and the pooled estimates by random effects meta-analysis (see section “main analyses” for details).

### Assessment of exposure to job strain

Job strain is self-reported in each of the studies listed in Table 1. The measure of job strain, i.e. the combination of high demands and low control, has previously been developed and harmonized, as documented by Fransson *et al.* ^16^, and applied in previous analyses on job strain and other outcomes, including health behaviours, cardiovascular disease and cancer ^8, 17– 20^. Briefly, study-specific measures for high demands and low control are defined dichotomously by the study-specific standardized median for each dimension (demands and control). Individuals with high demands and low control are considered exposed to job strain. The reference group is all other combinations of demands and control, i.e. individuals with low demands and high control, low demands and low control, high demands and high control.

### Assessment of covariates

Data on sex, age, cohabitation and SES will be included from each study to control for potential confounding influences. These covariates are chosen as potential confounders because they have been associated with depression ^21– 23^ and may be associated with job strain.

### Assessment of outcome

Diagnoses in the included hospital records are coded according to the International Classification of Disease (ICD) system ^24^ following versions 8, 9 or 10. Table 2 shows the diagnostic codes from each ICD-version we use to define unipolar depression. We include only principal diagnoses in the outcome definition, as auxiliary diagnoses may be underreported ^12^ and it is uncertain whether such underreporting is related to patient characteristics.

**Table 2.** Definition of outcome according to ICD-10, ICD-9 and ICD-8.

Classification	Codes	Name of codes
**ICD-10**	F32, F33	Depressive episode. Recurrent depressive disorder.
**ICD-9**	296.2, 296.3, 298.0, 311	Major depressive disorder, single episode. Major depressive disorder, recurrent episode. Depressive type psychosis. Depressive disorder not elsewhere classified.
**ICD-8**	296.09, 296.29, 298.09, 300.49	Involutional melancholia. Manic-depressive psychosis, depressed. Reactive depressive psychosis. Depressive neurosis.

## Main analyses

All study-specific analyses will be conducted using Cox proportional hazards regression with the occurrence of the first hospital record of unipolar depression as the failure-date, and censoring for migration (where available), death and end of follow up. All summary risk estimates will be calculated by pooling study-specific risk estimates and standard errors using inverse variance weighted random effects meta-analysis. Pooling will be conducted in R ( www.rproject.org) using the meta package ^25^ and the degree of heterogeneity between the study-specific estimates will be assessed by I ^2^ ^26^. Table 3 gives a ghost table for the main results.

**Table 3.** Ghost table for main results.

Study ^a^	Hazard ratio, job strain vs. no job strain ^b^	95% confidence interval	Heterogeneity (I ^2^)	P-value for I ^2^
**COPSOQ I**			-	-
**COPSOQ II**			-	-
**DWECS 2000**			-	-
**DWECS 2005**			-	-
**FPS**			-	-
**HeSSup**			-	-
**IPAW**			-	-
**PUMA**			-	-
**SLOSH 2006**			-	-
**SLOSH 2008**			-	-
**Still working**			-	-
**Whitehall II**			-	-
**WOLF-N**			-	-
**WOLF-S**			-	-
**Pooled estimate (random** **effects)**				

^a^Study acronyms: COPSOQ: Copenhagen Psychosocial Questionnaire Study, DWECS: Danish Work Environment Cohort Study, FPS: Finnish Public Sector study, HeSSUP: Health and Social support Study, IPAW: Intervention Project on Absence and Well-being, PUMA: Burnout, Motivation and Job Satisfaction study, SLOSH: Swedish Longitudinal Occupational Survey of Health, WOLF: Work, Lipids, Fibrinogen (N = Norrland, S = Stockholm).

^b^Hazard ratios are adjusted for sex, age and cohabitation.

### Confounder adjustment

The main analysis will be adjusted for sex, age and cohabitation. We will consider our hypothesis confirmed if the pooled adjusted hazard ratio is statistically significantly greater than 1 (p<0.05). We will not adjust for SES in the main analysis as this construct is conceptually intertwined with job strain ^27^, and consequently this model could then be considered overadjusted. However, analyses concerning whether the estimated risk is independent from SES will be included as a sensitivity analysis. Apart from cohabitation (self-reported, living with a partner/spouse, yes/no) the covariate measurements and categorizations have been documented previously ^8, 17– 19^. Briefly, SES will be based on occupation, except in data from the HeSSup study, where it will be based on highest educational qualification, and categorized as *low* (routine and manual occupations or comprehensive education), *intermediate* (non-manual intermediate occupations or vocational education), *high* (higher managerial, administrative and professional occupations or university-level education) or *other* (missing data on job title).

## Statistical power calculations

The expected numbers of cases of hospital-treated unipolar depression in each of the cohorts are presented in Table 4. The estimates are based on observations in the Danish register data concerning the incidence of hospital treatment for unipolar depression in gainfully employed Danes. These numbers were applied to the studies to estimate the expected number of cases, and the observed numbers of cases in the databases may diverge from this estimation. If there are no observed cases amongst individuals exposed to job strain in a study, this study will not be included in the analyses, as a risk estimate cannot be obtained.

**Table 4.** Study-specific expected number of hospital diagnosed cases of unipolar depression.

Study ^a^	Approximate length of follow up, years	Expected cases of unipolar depression
**COPSOQ I**	10	25
**COPSOQ II**	5	24
**DWECS 2000**	10	80
**DWECS 2005**	5	29
**FPS**	10	692
**HeSSup**	10	239
**IPAW**	10	30
**PUMA**	10	25
**SLOSH 2006**	5	37
**SLOSH 2008**	5	43
**Still working**	10	133
**Whitehall II**	10	150
**WOLF-N**	10	68
**WOLF-S**	10	83
**Total (sum)**		1659

^a^Study acronyms: COPSOQ: Copenhagen Psychosocial Questionnaire Study, DWECS: Danish Work Environment Cohort Study, FPS: Finnish Public Sector study, HeSSUP: Health and Social support, IPAW: Intervention Project on Absence and Well-being, PUMA: Burnout, Motivation and Job Satisfaction study, SLOSH: Swedish Longitudinal Occupational Survey of Health, WOLF: Work, Lipids, Fibrinogen (N = Norrland, S = Stockholm).

**Figure 1.**
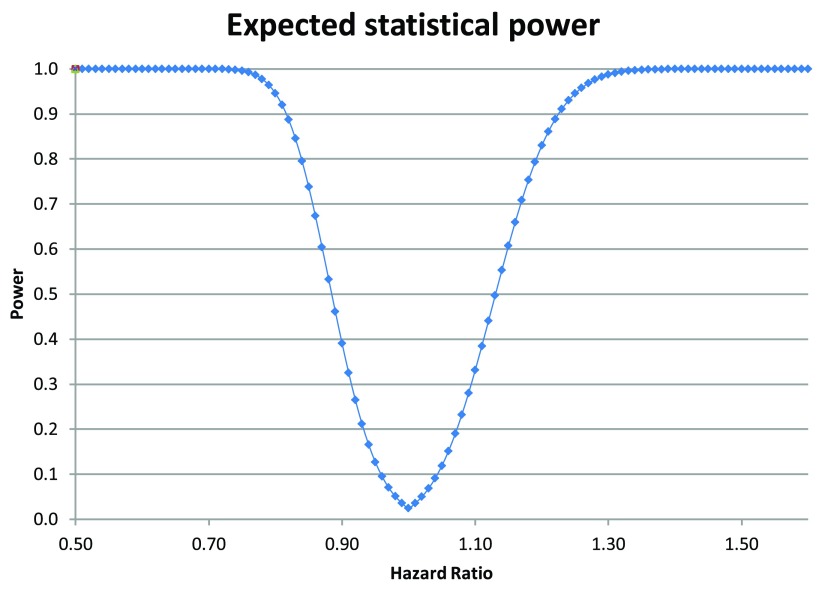
Expected statistical power as a function of the hazard ratio.

The expected statistical power as a function of the hazard ratio is shown in Figure 1. The planned analysis is expected to be powered to show an association of 1.23 with >90% power. This is under the assumption that the actual number of cases will match the expected number of cases in Table 4 and that all studies will provide cases and thus are included in the analysis.

## Sensitivity analyses

The following section specifies the planned sensitivity analyses which will examine the robustness of the results. All statistical tests for the sensitivity analyses will be two-sided with a significance threshold of P<0.05. We will conduct a series of sensitivity analyses which may lead to concerns of mass significance due to multiple testing. To avoid inflating the type I error due to multiple testing, we will consider the sensitivity analyses nested within the main hypothesis test. Thus, their interpretation will depend on the results of the main analysis: if our main hypothesis is confirmed, we will consider the significance tests of the nested hypotheses valid and the tests which yield p-values <0.05 statistically significant. The sensitivity analyses may in this case be considered as an examination of the conditions under which the average population effect found in the main analysis holds. However, if the main hypothesis is not confirmed, we will not consider the tests of the sensitivity analyses (nested hypotheses) with p<0.05 confirmatory, i.e. the results of the sensitivity analyses will be considered exploratory and hypothesis generating. This strategy will retain the overall probability of a type I error under 0.05, whenever the main null hypothesis is true.

Our first set of sensitivity analyses examines whether the association between job strain and depression is modified by sex, age (≤35, 36–49, 50+ years) or SES (low, intermediate, high ^8^) following indications from previous studies ^28– 30^. If there are too few cases to obtain estimates for 3 categories of age and SES in more than half of the eligible studies, we will use the categories ≤49/50+ years and low SES/other instead. Following the STROBE recommendations ^31^ we will present results on effect-modification so that both departure from additivity and multiplicativity may be assessed ^32^. However, our conclusions on effect-modification will be based on departure from additivity, as such information is particularly important from clinical and public health perspectives ^31, 33, 34^. The statistical test will be based on the Central Limit Theorem, and Gauss propagation of error formulas.

If any statistically significant associations are found in the main analyses, we will conduct a second set of sensitivity analyses to examine how these associations are affected by accounting for SES, self-reported baseline mental health, and chronic physical disorders. Regarding mental health, we will a) adjust for mental health (continuous score, see Table 5 for measures) and b) exclude individuals with poor mental health (the poorest quintile, based on the measures presented in Table 5). Regarding chronic physical disorders we will exclude individuals with baseline coronary heart disease, stroke, cancer, chronic obstructive pulmonary disease, musculoskeletal disorders or diabetes, where data are available, as chronic physical disorders are associated with increased risk of mental disorder ^35^. In a third and final sensitivity analysis we will examine how unipolar depression is related to the separate dimensions of demands and control (standardized continuous scores and the job strain model quadrants, i.e. comparing the three other combinations of demands and control to individuals with low demands and high control (cf. ^8^).

**Table 5.** Measures of mental health at baseline.

Study ^a^	Mental health at baseline	Source (reference)
**COPSOQ I**	Mental health inventory (MHI-5)	The Short Form Health Survey ^36^
**COPSOQ II**	Depressive symptoms	The Copenhagen Psychosocial Questionnaire II ^37^
**DWECS 2000**	Mental health inventory (MHI-5)	The Short Form Health Survey ^36^
**DWECS 2005**	Mental health inventory (MHI-5)	The Short Form Health Survey ^36^
**FPS**	General Health Questionnaire (GHQ-12)	General Health Questionnaire ^38^
**HeSSup**	Depressive symptoms	Beck depression inventory ^39^
**IPAW**	Mental health inventory (MHI-5)	The Short Form Health Survey ^36^
**PUMA**	Mental health inventory (MHI-5)	The Short Form Health Survey ^36^
**SLOSH 2006**	Depressive symptoms	Symptom Check List, 6 item subscale ^40, 41^
**SLOSH 2008**	Depressive symptoms	Symptom Check List, 6 item subscale ^40, 41^
**Still working**	Not available	-
**Whitehall II**	Depressive symptoms (GHQ-30)	General Health Questionnaire ^38^
**WOLF-S**	Not available	-
**WOLF-N**	Not available	-

^a^Study acronyms: COPSOQ: Copenhagen Psychosocial Questionnaire Study, DWECS: Danish Work Environment Cohort Study, FPS: Finnish Public Sector study, HeSSUP: Health and Social support Study, IPAW: Intervention Project on Absence and Well-being, NWCS: The Netherlands Working Conditions Survey, POLS: Permanent Onderzoek Leefsituatie, PUMA: Burnout, Motivation and Job Satisfaction study, SLOSH: Swedish Longitudinal Occupational Survey of Health, WOLF: Work, Lipids, Fibrinogen (N = Norrland, S = Stockholm).

## Discussion

A major strength of the planned analyses is the register-based outcome with a specific diagnosis. The diagnoses are based on clinical assessments, which are independent of job strain assessments, and have high validity ^42^. The use of such outcomes has not been possible in most previous analyses on work environment and depression ^9, 10^, as the relatively low incidence of hospital-treated unipolar depression necessitates an exceptionally large dataset. Some exclusively register-based analyses (e.g. ^43^) have been conducted previously using hospital discharge registers. Such analyses have, however, not examined work environment variables measured at the individual level but exposures approximated using job exposure matrices. Although such approximation is useful when exposure measures are unavailable, the lack of individual level measurement hampers the interpretation of the findings as they are open to the ecological fallacy ^44^.

Measuring depression incidence exclusively through hospital treatment registers also has limitations. It is likely that only the most severe cases of depression are treated in a hospital. Consequently, the results of the planned analyses will not be extendable to mild and moderate depression, and severe depression not treated in a hospital, if the aetiology of depression varies with severity and treatment. Furthermore, the exclusion of individuals with depression before or at baseline, to ensure a prospective study design, is also based on hospital-treated depressive episodes. Hence, it is possible that participants are suffering from, or have suffered from, untreated depression before baseline. However, in this consortium study, the only way to obtain accurate retrospective diagnoses is based on hospital treatment. Although data on treatment with antidepressants for example may also be obtained from registers, these medications are used to treat a range of conditions, other than depression, such as anxiety, pain and incontinence ^45^, and consequently do not provide any accurate diagnostic information.

Another potential limitation of the planned analyses is the self-reported exposure data which could be sensitive to reporting biases due to negative affect at baseline; a bias which may result in inflated risk estimates, if individuals with reduced mental wellbeing report their exposure more negatively and are at increased risk of developing depression ^46^. We address this limitation via the sensitivity analyses by adjusting for baseline mental health. Although this analysis may be overadjusted, at least if negative affect at baseline is a consequence of the working environment, any remaining association between job strain and depression will provide a strong argument that the results are not explained by reporting bias.

The planned analysis uses data from 14 studies conducted in 4 European countries. The studies differ in design, timing, and study population. Whereas some studies (e.g. DWECS, SLOSH) include the general working population, others are restricted to employees of specific organisations or occupations (e.g. FPS, Whitehall). Although this combination of different study populations means great gains in statistical power, the generalizability of the findings must be considered. Ideally, we may generalize our findings to the working population of (Northern) Europe. Such generalization would be supported by low degrees of heterogeneity in the pooled estimates, as we found for the association between job strain and coronary heart disease ^8^. If there are greater levels of heterogeneity in the findings, however, the generalizability of the associations outside the examined cohorts is less clear. In that case we may need additional post hoc sensitivity analyses to examine the reasons for heterogeneity, for example the length of follow up, the period with hospital data available before baseline, and whether or not outpatient hospitalisation data are included throughout the follow up period.

All participating studies have been approved separately by the relevant national ethical committees (see appendices of ^8, 17^ for details). The results of this planned study will be published in an article in a scientific peer-reviewed journal. This planned study will constitute the largest in the field to date and as such is likely to set the parameters of research in this field for some time to come.

## Project organization

The project is organized as part of the IPD-Work (“Individual-participant-data meta-analysis of working populations”) Consortium ^47^. IPD-Work was funded by the NEW OSH ERA ^48^, and is led by Professors Mika Kivimäki (Finnish Institute of Occupational Health and University College London, UK), Töres Theorell (Stress Research Institute, University of Stockholm, Sweden), Reiner Rugulies (National Research Centre for the Working Environment, Denmark) and Nico Dragano (Department of Medical Sociology, University of Düsseldorf, Germany). The IPD-Work Consortium is coordinated by Prof. Mika Kivimäki. The principal investigator in the analyses described in the present protocol is Dr. Ida E. H. Madsen, researcher at the National Research Centre for the Working Environment, Denmark.
