# Supplementary material for: Study protocol for examining job strain as a risk factor for severe unipolar depression in an individual participant meta-analysis of 14 European cohorts
Source: F1000Res. 2014 Feb 26;2:233. Originally published 2013 Nov 5. [Version 2] doi: 10.12688/f1000research.2-233.v2 (PMC3938244; doi:10.12688/f1000research.2-233.v2)
Supplement: Peer review history

# Peer review: Referee response for version 2

**DOI:** 10.5256/f1000research.3914.r3895

**Published:** 2014-03-26

**Authors:** Pamela L. Perrewé

**Affiliations:** 1 Department of Management, Florida State University, Tallahassee, FL, USA

I have read this submission. I believe that I have an appropriate level of expertise to confirm that it is of an acceptable scientific standard.

# Peer review: Referee response for version 1

**DOI:** 10.5256/f1000research.2555.r3029

**Published:** 2014-01-27

**Authors:** Pamela L. Perrewé

**Affiliations:** 1 Department of Management, Florida State University, Tallahassee, FL, USA

I find the proposal examining the association between high strain jobs and unipolar depression to be fascinating.  Examining clinically diagnosed depression as opposed to self-reported depression is an important step in job strain research.  My only suggestion is to be cautious about arguing that individuals with high strain jobs leads to depression. This is not a longitudinal study, and it is just as likely that individuals who suffer from clinical depression are more likely to hold jobs that are characterized by high demands and low control. Regardless, this is an important piece of research and I look forward to reading the findings.

I have read this submission. I believe that I have an appropriate level of expertise to confirm that it is of an acceptable scientific standard.

# Peer review: Referee response for version 1

**DOI:** 10.5256/f1000research.2555.r2772

**Published:** 2013-12-16

**Authors:** Joanna Wieclaw

**Affiliations:** 1 MarselisborgCentret, Aarhus, Denmark

**Regarding the title**

I find it a bit misleading that the title says “14 European cohorts” as the study utilizes the Scandinavian cohort and only one other cohort, the UK's Whitehall study.

**Article content**

As the authors themselves point out, the studies included differ in design, timing and study population, which consist a challenge for a meta-analyses. The authors propose a number of sophisticated statistical methods to meet this challenge. However I have some reservations, particularly about including the Whitehall study - which is otherwise very good - as it is the only non Scandinavian study, and includes a selected population of white collars employees in a specific environmental setting. Also, to my knowledge, the UK registers differ from the Scandinavian practices, which renders comparisons problematic.

Regarding measure of job strain it is not quite clear to me if additive, multiple, or both conceptualisation will be used. Also I find using a dichotomous measure of variables involved (low/high) rather crude. I suggest underlining more clearly that the risk of depression will be calculated in relation to other possible combinations of job strain model variables, which I find important as there is some evidence of diverging results.

The measure of SES based only on occupation is a bit limited. Is it not possible to include fx. income and urbanity?

The description of the statistics is detailed and lengthy. For the readers with scientific background this is very satisfactory, but I believe that the readers with less research training will be lost here. I suggest that the purpose of this sophisticated statistical analyses be more clear, or may be also plainly explained.

Is it necessary to go as far back as ICD-8 in obtaining cases? And is “manic-depressive psychosis” included? I believe it would make data unnecessary heterogeneous.

**Conclusion**

In all I find the study interesting and well designed. Its strength are the large population and register-based outcome. At the same time the limitation is that the outcome is restricted to a rather severe case of depression, and only ones treated in psychiatric hospitals, which are less prevalent.

I have read this submission. I believe that I have an appropriate level of expertise to confirm that it is of an acceptable scientific standard.
